# Co-Infection of Chickens with *Staphylococcus lentus* and *Staphylococcus aureus* from an Outbreak of Arthritis, Synovitis, and Osteomyelitis Argues for Detailed Characterisation of Isolates

**DOI:** 10.3390/ani14172574

**Published:** 2024-09-04

**Authors:** Miguel Matos, Peter Mitsch, Dieter Liebhart, Michael Hess, Claudia Hess

**Affiliations:** 1 Clinic Unit for Poultry Medicine, Clinical Center for Population Medicine in Fish, Pigs, and Poultry, University of Veterinary Medicine, Veterinaerplatz 1, 1210 Vienna, Austria; dieter.liebhart@vetmeduni.ac.at (D.L.); michael.hess@vetmeduni.ac.at (M.H.); claudia.hess@vetmeduni.ac.at (C.H.); 2 Tierarzt GmbH Dr. Mitsch, Hauffgasse 24, 1110 Vienna, Austria; mitsch@mitsch.co.at

**Keywords:** organic broiler breeder flock, antibiotic resistance, MALDI-TOF MS, joint inflammation, co-infection, bacterial characterisation, poultry health

## Abstract

**Simple Summary:**

This manuscript reports on an outbreak of joint infections in an organic broiler breeder flock in Austria, associated with two types of bacteria: *Staphylococcus aureus* and *Staphylococcus lentus*. The clinical picture of the affected chickens included weakness, lethargy, and difficulty walking, with some birds succumbing to the infection. Detailed examinations revealed severe joint inflammation and damage. Laboratory analyses confirmed the presence of both bacteria, with tests showing resistance to many antibiotics. This study underscores the importance of recognising and understanding less common bacteria like *S. lentus*, which are not frequently reported but can seriously impact poultry health. The findings highlight the need for thorough bacterial identification in outbreaks to improve disease management and prevention strategies.

**Abstract:**

*Staphylococcus* species are widespread in poultry environments and can cause various infections, often when the host’s defences are compromised. This manuscript reports on a co-infection of chickens with *Staphylococcus lentus* and *Staphylococcus aureus* associated with an outbreak of arthritis, synovitis, and osteomyelitis in an organic broiler breeder flock in Austria. Clinically, the affected flock showed weakness, lethargy, lameness, and increased mortality. Post-mortem examinations identified purulent arthritis and femoral head necrosis. Bacteriological analysis using MALDI-TOF MS identified both *S. aureus* and *S. lentus* in the affected joints. Antibiotic resistance testing revealed significant resistance, particularly in *S. lentus*. Histological analysis showed severe inflammation and bacterial colonies in the joints. While *S. aureus* is a common pathogen in poultry, *S. lentus* is less frequently reported. This study emphasises the need for detailed bacterial characterisation in outbreaks to better understand the role of less common pathogens like *S. lentus*. Further research is necessary to elucidate the impact of *S. lentus* on poultry health and its role in causing arthritis and synovitis, highlighting the importance of comprehensive investigation in such outbreaks.

## 1. Introduction

*Staphylococcus* encompasses several species and subspecies, making it the most prominent genus in the Staphylococcaceae family [1]. These bacteria are Gram-positive, coccoid, and typically cluster together when grown on solid media. Under aerobic conditions, *S. aureus* forms circular, smooth, β-haemolytic colonies, measuring 1–3 mm in diameter, with pigmentation that often ranges from white to orange.

*Staphylococcus* species are widespread, commonly inhabiting the skin and mucous membranes, and are prevalent in environments where poultry are bred, raised, or processed. Various *Staphylococcus* species, including *S. aureus*, *S. epidermidis*, *S. xylosus*, *S. cohnii*, *S. lentus*, *S. saprophyticus*, *S. sciuri*, and *S. gallinarum*, have been isolated from the skin and nasal passages of healthy poultry [2]. Infections often occur when the host’s natural defences are compromised, such as through skin injuries, mucous membrane inflammation, parasitic infections, immunosuppressive conditions, or unhealed navel infections in newly hatched chicks [2,3]. Clinically, these infections can result in yolk sac infections during the first week of life and osteomyelitis lesions in the bones of older birds. Osteomyelitis lesions consist of focal yellow areas of caseous exudate or lytic areas, making affected bones fragile. The proximal tibiotarsus and proximal femur are most frequently involved, with affected birds often experiencing disarticulation and/or fracture of the coxofemoral joint and femoral head necrosis. Arthritis, periarthritis, and synovitis are also common, with affected joints becoming swollen and filled with inflammatory exudate as osteomyelitis extends from nearby metaphyseal areas [4]. This report describes an outbreak of arthritis with synovitis and osteomyelitis in an organic broiler breeder flock in Austria associated with *S. aureus* and *S. lentus*.

## 2. Materials and Methods

### 2.1. Case Description

In the federal state of Upper Austria, Austria, a 35-week-old organic broiler breeder flock of 5450 Hubbard hens and 550 cockerels, was housed in a barn system with deep litter bedding and adhered to a standardised vaccination regimen. The flock had a history of a colibacillosis outbreak at 26 weeks old, resulting in a mortality rate of 1.7%, which was initially stabilised with enrofloxacin treatment. However, in the subsequent weeks, the whole flock was affected, with enrofloxacin failing to yield therapeutic effects. The clinical presentation included weakness, lethargy, lameness, and occasionally cannibalism, culminating in heightened mortality rates. Over time, the clinical condition transitioned into a chronic state, marked by the daily loss of 1–2 birds and a lack of uniformity within the flock. Morbidity was estimated at approximately 5%. Despite these challenges, production data indicated an 85% laying rate, with 80% of eggs being fertilised and maintaining good egg quality.

### 2.2. Post-Mortem Investigation, Bacterial Isolation, and Identification

Three chickens were referred to the Clinic for Poultry Medicine at the University of Veterinary Medicine Vienna, Vienna, Austria, for further examination. Necropsies were conducted according to standard procedures, and bacteriological investigation smears were obtained from the heart, liver, intestine, and hock joints. Specimens were directly plated onto Columbia agar supplemented with 5% sheep blood (COS; bioMerieux, Vienna, Austria), MacConkey agar (Scharlau, Vienna, Austria), and Schaedler agar containing 5% sheep blood (SCS; bioMerieux). COS and MacConkey agar plates were aerobically incubated at 37 °C, while SCS plates were anaerobically incubated (Genbox anaer; bioMerieux) at 37 °C for 24 h.

Identification of bacterial isolates was performed using Matrix-Assisted Laser Desorption/Ionisation–Time of Flight Mass Spectrometry (MALDI-TOF MS) (Microflex LT instrument, Bruker Daltonics GmbH, Bremen, Germany) using the Bruker Daltonic reference library version 4.1.80, following the manufacturer’s guidelines. Sample preparation was conducted according to the manufacturer’s guidelines for the extraction method using formic acid and acetonitrile.

### 2.3. Antibiotic Susceptibility Testing

An antibiogram was generated using an agar diffusion test, following the Clinical and Laboratory Standards Institute (CLSI) guidelines as outlined in the 5th edition of the Performance Standards for Antimicrobial Disk and Dilution Susceptibility Tests for Bacteria Isolated From Animals [5]. The evaluation of the agar diffusion test involved measuring the inhibition zone diameters in millimetres using a ruler. These measurements were then compared to the appropriate critical diameter table. The categorisation of the bacterial response to the antibiotics as “sensitive”, “intermediate”, or “resistant” was determined following the CLSI standards.

### 2.4. Histology

During necropsy, samples from the hock joints were taken, fixed in Formical-4™ Decalcifier (StatLab Medical Products, McKinney, TX, USA), and subsequently embedded in paraffin blocks. Tissue sections, measuring 4 µm in thickness, were cut using a microtome (Microm HM 360; Microm Laborgerate GmbH, Walldorf, Germany), affixed to glass slides, and stained with hematoxylin and eosin (H&E).

## 3. Results

### 3.1. Necropsy, Bacterial Isolation, and Identification

**Figure 1:**
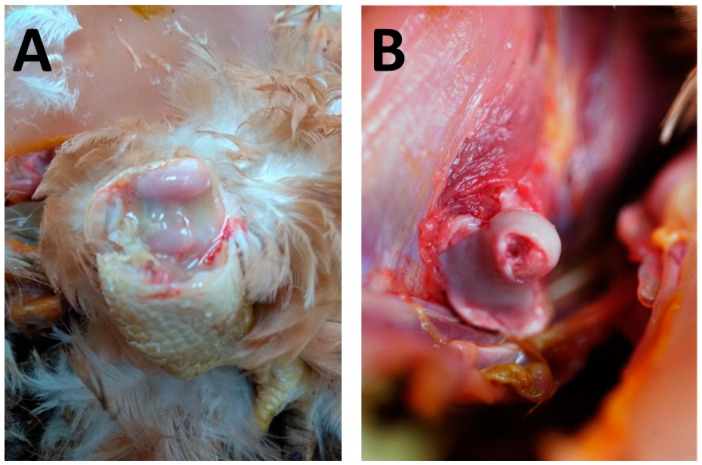
Main pathological findings observed during the necropsy: arthritis of the hock joints, characterised by swelling, and yellow exudate involving the tendon sheaths in the epiphysis of tibiotarsus and tarsometatarsus (tenosynovitis) (**A**); chondronecrosis and osteomyelitis of the proximal femur (**B**).

The chickens subjected to post-mortem examination were exclusively female birds, with body weights of 1.1 kg, 1.2 kg, and 1.8 kg, respectively. Feathering around the pericloacal region was soiled with faecal matter, and two of the birds exhibited shortened tibiotarsi. Additionally, these individuals displayed purulent arthritis in the hock joints and necrosis of the femoral heads (Figure 1).

A substantial number of *Staphylococcus* spp. colonies were isolated from the hock joints in COS agar, with lower counts detected in samples from the heart and liver. Further characterisation through MALDI-TOF MS analysis revealed *Staphylococcus aureus* and *Staphylococcus lentus* in the hock joints (Table 1). Detailed findings from the antimicrobial resistance testing are outlined in Table 2.

### 3.2. Histology

**Figure 2:**
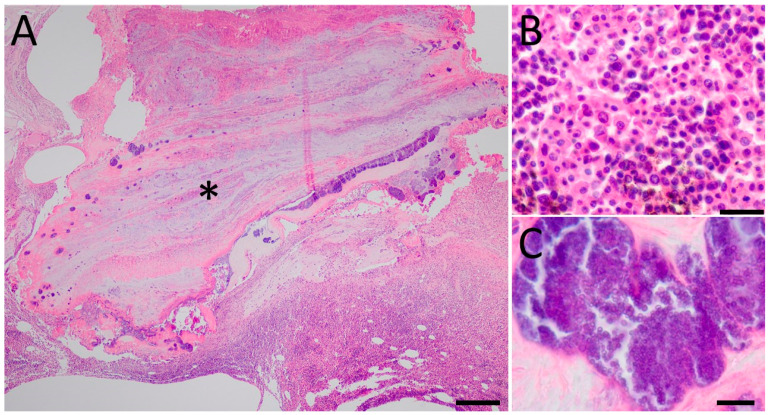
Histological investigation of the affected hock joints. (**A**) a tendon (*) showing severe fibrinoheterophilic inflammation (highlighted in **B**) associated with numerous colonies of bacteria of coccoid morphology (highlighted in **C**). Haematoxylin and eosin (H&E) staining. Bar = 200 µm (**A**), 25 µm (**B**), 10 µm (**C**).

Joints showed massive accumulations of heterophilic granulocytes and necrotic material in the joint space. In addition, numerous colonies of coccoid bacteria could be seen in the lesions and the surrounding tendons (Figure 2).

## 4. Discussion

Although various *Staphylococcus* spp. can be implicated in different pathological conditions in poultry, some are more frequently associated with specific infections. For instance, *S. aureus* is commonly linked to bumblefoot and gangrenous dermatitis, while *S. hyicus* is often found in cases of turkey stifle joint osteomyelitis, eye infections (such as blepharitis and conjunctivitis), and acantholytic dermatitis, and *S. simulans* and *S. agnetis* are typically associated with endocarditis [4]. Even though a wide range of *Staphylococcus* spp. have been isolated from cases of arthritis, *S. aureus* has been the most commonly identified and is thus considered to be the *Staphylococcus* of biggest health concern in poultry [4]. Nonetheless, when conducting differential diagnosis investigations for arthritis and synovitis in chickens, it is essential to consider other conditions that mimic staphylococcosis, such as infections caused by *Escherichia coli*, *Pasteurella multocida*, *Salmonella gallinarum*, *Mycoplasma synoviae*, reoviruses, and bone disorders resulting from mechanical trauma. In this report, both gross and histopathological examinations confirmed a bacterial nature of the outbreak. Further bacteriological analyses, including MALDI-TOF, identified two staphylococcal species associated with the condition: *S. aureus* and *S. lentus*. While *S. aureus* is coagulase-positive, most other staphylococci found in poultry, including *S. lentus*, are coagulase-negative [2]. Although biochemical panel tests can distinguish these bacteria, protein fingerprinting using MALDI-TOF is a powerful method for identifying and differentiating *Staphylococcus* species [6,7], including coagulase-negative staphylococci [8]. While coagulase-positive staphylococci, like *S. aureus*, are primary health concerns in poultry, infections by coagulase-negative staphylococci (CNS), such as *S. lentus*, are also documented in cases of arthritis and synovitis, though less frequently [4,9]. *S. lentus* was the most prevalent CNS species in Belgian poultry farms in the 1970s [10] and recently ranked as the third most common *Staphylococcus* species in Western Poland’s poultry farms (13.9%), being the most prevalent in breeding hens (31.1%) [11]. Over 80% of isolated Staphylococci in the latter study were CNS. Comprehensive investigations indicate that CNS can cause persistent infections and produce various virulence factors, though the specific role of these factors in pathogenicity remains unclear [12,13]. It is crucial to conduct additional research to clarify the role of *S. lentus* in such outbreaks, investigating in which way, and if at all, it is a contributing cause of arthritis and synovitis in chickens. 

Outbreaks of severe arthritis and synovitis in chickens caused by *Staphylococcus* infections can arise suddenly and pose significant health problems. These infections typically occur when the host’s natural defences are breached, often through skin wounds or inflamed mucous membranes, leading to osteomyelitis in the metaphyseal joints [2]. Joint and tendon sheath lesions are often linked to concurrent bone changes [14,15]. Thus, the affected flock’s prior colibacillosis infection weakened its immunity and increased its susceptibility to staphylococcosis. This condition resulted in reduced activity, decreased feed consumption, and higher mortality rates. Opportunistic *Staphylococcus* spp. exploited these vulnerabilities, further aggravated by stress and cannibalism within the flock, with chronically affected birds exhibiting swollen joints, reluctance to move, and debilitation. Severe issues with hock joint synovitis in breeder flocks have been observed across all ages, with a notable increase during the second half of the rearing period, post-transport, and at the onset of production, likely due to oestrogenic and immunosuppressive effects [16].

There is currently no effective vaccine for *S. aureus*, and staphylococcal bacterins have proven ineffective in preventing poultry infections [2,3]. Management relies on antibiotic therapy, but treating localised lesions is challenging due to poor antibiotic penetration [17], which is additionally discouraged in organic farming. Chronic infections are particularly difficult to treat as necrotic foci in bones hinder antibiotic effectiveness [17]. Sick birds often struggle to eat or drink, reducing drug intake. Additionally, antibiotic resistance is of extreme importance in isolated *Staphylococcus* strains; thus, sensitivity tests should always be performed. In the present study, *S. lentus* showed resistance to important therapeutic drugs such as tetracycline and spectinomycin. Similarly, resistance to tetracyclines, penicillins, and macrolides was a common resistance phenotype in isolated *S. lentus* from chickens and turkeys in Poland [18]. It has been suggested that CNS strains present higher resistance levels to antibiotics than *S. aureus* and that multi-resistant CNS are observed with increased frequency [11,18,19]. In general, in our investigation, *S. aureus* was more susceptible to the tested antibiotics, including enrofloxacin, which was previously used in the flock. More studies are necessary to determine the impact of commensal CNS strains, which can act as reservoirs and sources of antibiotic-resistant genes, on the health of poultry species.

Organic farming discourages antibiotic use, emphasising preventive measures [20]. Effective management includes practices that reduce damage to host defences, such as minimising wounds, stress, and other diseases. Preventing injuries is crucial since wounds are entry points for *S. aureus*. Implementing biosecurity measures and proper vaccination schemes are also essential for minimising leg problems.

## 5. Conclusions

In conclusion, this report presents a case of staphylococcal arthritis with synovitis and osteomyelitis in an organic broiler breeder flock, exacerbated by previous conditions. Bacterial identification by MALDI-TOF investigation revealed the presence of *S. lentus*, in addition to *S. aureus*, associated with the outbreak. *S. aureus* is recognised as a significant opportunistic pathogen in poultry and is the most frequently reported *Staphylococcus* spp. associated with clinical disease in the field. However, further research is necessary to elucidate the role of *S. lentus* as a causative agent of arthritis and synovitis in chickens.

## Figures and Tables

**Table 1:** Log (scores) of *Staphylococcus* isolates investigated by MALDI-TOF, based on the Bruker Daltonic database.

Sample	Matched Pattern	Log (Score) ^a^
PA18_25301 A	*Staphylococcus lentus* LMG 19120 LMG_corr	2.14
PA18_25301 B	*Staphylococcus aureus subsp. aureus* DSM 799 DSM	2.61

^a^ A log(score) ranging from 1.7 to 2.0 indicates genus-level identification; a log(score) exceeding 2.0 corresponds to species-level identification. Any log(score) below 1.7 is considered unidentifiable by the software.

**Table 2:** Antibiogram results of the *Staphylococcus aureus* and *Staphylococcus lentus* isolates identified by MALDI-TOF. R: resistant; I: intermediate; S: susceptible.

	*S. aureus*	*S. lentus*
Oxalic acid (2 µg)	R	R
Spectinomycin (100 µg)	S	R
Tilmycosin (15 µg)	S	I
Trimethropin/Sulfamethoxazol (1.25/23.7 µg)	R	I
Tylosin (15 µg)	S	I
Amoxicillin (10 µg)	I	I
Ampicillin (10 µg)	I	I
Colistin (10 µg)	R	R
Doxycycline (30 µg)	I	S
Enrofloxacin (5 µg)	S	I
Neomycin (30 µg)	I	I
Tetracycline (30 µg)	I	R

## Data Availability

Data is contained within the article. The original contributions presented in the study are included in the article, further inquiries can be directed to the corresponding author/s.
